# The ‘side effects’ of digitalization: A study on role overload and job burnout of employees

**DOI:** 10.1371/journal.pone.0322112

**Published:** 2025-04-30

**Authors:** Quanjun Zhang, Wei Dai, Jian Chen, Yuan Gu, Yang Zhao

**Affiliations:** 1 Guangxi Big Data Research Institute, Nanning, Guangxi, China; 2 Institute of Science, Technology and Industrial Development, Sichuan College of Architectural Technology, Deyang, Sichuan, China; 3 Department of Geography, University College London, London, England, United Kingdom; 4 Department of Public Administration and Humanities, Dalian Maritime University, Dalian, Liaoning, China; 5 School of Law, Southwest University of Science and Technology, Mianyang, Sichuan, China; International University - Vietnam National University Ho Chi Minh City, VIET NAM

## Abstract

In the context of the digital economy, enterprise digitalization represents a significant opportunity to enhance core competitiveness. Nevertheless, the digitalization of enterprises may have unanticipated effects on employee’s mental health, which could impede the digital advancement of the enterprise. Such consequences may be evidenced by employee role overload and burnout, which in turn impede enterprise digitalization. This study employed the JR-D model to investigate the empirical data obtained from 250 completed questionnaires by employees. The results indicated that (1) enterprise digitalization had a positive effect on young employees’ levels of role overload and burnout; (2) employees’ role overload served as a mediator between enterprise digitalization and burnout; (3) perceived organizational support moderated the direct predictive effect of enterprise digitalization on burnout and the indirect predictive effect of enterprise digitalization on burnout through role overload, respectively. The higher the level of perceived organizational support, the weaker the direct and indirect predictive effects would be. This study proposed measures and recommendations to mitigate the unintended consequences of digitalization and promote optimal advancement of enterprise digitalization.

## 1. Introduction

The advent of the digital economy has seen the penetration of digital technologies such as big data, artificial intelligence, the internet of things, and cloud computing. These technologies are enabling the traditional real economy to unleash enormous economic potential. In the digital economy, enterprises are utilizing cloud computing, artificial intelligence, blockchain, and other digital tools to carry out a variety of business scenarios. This is prompting enterprises to reshape and transform their organizational structure, business processes, and business models at multiple levels and in various directions [1]. Enterprise digitalization can be defined as a form of organizational change that enables enterprises to adapt to the digital economy.

Contemporary research on digital phenomena frequently employs the terms digitization, digitalization, and digital transformation, each delineating distinct yet interrelated concepts. Digitization denotes the technical process of converting analog data, such as physical documents or media, into digital formats, facilitating improved storage, retrieval, and processing [2]. Digitalization extends beyond this foundational step, encompassing the integration of digital technologies into operational processes to enhance efficiency, connectivity, and data-driven decision-making [3]. Digital transformation, in contrast, signifies a more expansive shift, involving a strategic, organization-wide reconfiguration that leverages digital innovations to redefine business models, customer interactions, and value propositions [4]. Despite these distinctions in scope and application, the conceptual underpinnings of these terms converge on the centrality of digital technologies in enabling change, whether at a technical, operational, or strategic level [3]. By centering on digitalization, this research bridges the technical groundwork of digitization and the broader implications of transformation, maintaining a consistent analytical framework suited to its objectives.

The China Academy of Information and Communications Technology defines enterprise digitalization as the process of leveraging digital technology to digitize all elements and links of an enterprise, thereby facilitating the reorganization and transformation of business processes and production methods. This approach aims to enhance economic efficiency, reduce operating costs, and facilitate the transformation and upgrading of traditional models. From the perspective of organizational change cognition, when external environmental factors impact an organization, it will adjust its internal conditions to maintain equilibrium between the actual needs and conditions of its inner environment and the external environment, thereby promoting its survival and development [5]. Digitalization introduces significant strategic and operational changes within organizations, presenting opportunities and challenges, which can disrupt established practices and require effective management strategies for successful integration [6].

Digitalization is often touted to optimize knowledge usage, thereby boosting productivity and efficiency. However, from employees’ perspectives, these expectations may not materialize, and the consequences of digitalization can even be detrimental [7]. As digitalization deepens and digital technology is increasingly applied, enterprises are placing heightened expectations on employee work efficiency and quality. The psychological and physical costs associated with the enhancement of job requirements are likely to have a detrimental impact on employees’ mental health. In comparison to their more experienced counterparts, younger employees are frequently overwhelmed psychologically and are more prone to feelings of anxiety and burnout when confronted with occupational, social, personal, and interpersonal pressures [8]. For further exploration, we introduce the Job Demands-Resources Model (JD-R model), a well-established framework that explains how job demands and job resources interact to influence employee well-being and performance [9]. The model categorizes occupational circumstances as either job demands or resources [10]. Job demands are defined as the physical and mental resources that are expended in the course of performing job-related tasks. Job demands can result in the expenditure of work-related physical or psychological costs, which may subsequently lead to a reduction in engagement, an increase in burnout, and a decline in work output. Conversely, job resources have the potential to enhance resource input, mitigate work-related physical and psychological costs, and mitigate the adverse effects of job demands [11]. In the digital age, job burnout and role overload present novel characteristics. From the JD-R perspective, the rise of digitalization has significantly altered both job demands and job resources. For example, the pervasive culture of constant availability exacerbates burnout. The digital age has increased work permeation through telecommuting, instant messaging, and social media, which has blurred the boundaries between work and life, making it difficult for employees to completely disconnect from work and leading to chronic fatigue [12]. Furthermore, digital surveillance pressure has intensified. Organizations are increasingly adopting digital monitoring tools, such as AI performance assessments and remote work monitoring, which have led to employees feeling more external control and a lack of autonomy [13]. Role overload refers to the fact that individuals face too many tasks in a limited time, and this problem is more prominent in the era of digitalization. Information overload brought by digitalization exacerbates role conflict, and excessive information input leads to cognitive overload, which exacerbates role conflict [14]. According to the JD-R theory, role overload can be viewed as a job demand that depletes employees’ cognitive and emotional resources, heightening stress and reducing their ability to engage productively at work. There is a great deal of role ambiguity in the Platform Economy. For example, telecommuters, self-employed, and freelancers are taking on multiple roles on digital platforms, such as the increase in ‘slash workers’ [15]. JD-R theory posits that role ambiguity reduces the availability of job resources, making it difficult for employees to manage competing demands effectively, thereby increasing psychological strain and burnout. Role ambiguity makes it difficult for individuals to distinguish between formal and informal tasks, increasing role load [16]. Nevertheless, there has been a paucity of research on the occupational anxiety and job burnout under the influence of digitalization over the past five years. In light of this, this study seeks to elucidate the impact of enterprise digitalization on role load and job burnout of employees in accordance with the JD-R model. In the digital context, comprehensive investigation of the occupational psychological status of workers can mitigate job burnout and enhance their work attitude and mental health.

## 2. Literature review and hypotheses

Digitalization refers to the adoption and integration of digital technologies into organizational processes, products, and services, aiming to enhance operational efficiency, value creation, and innovation [17]. Enterprise digitalization can be defined as a business transformation, a profound transformation and reconstruction of business, management, and business model driven by a new generation of ICT technology [18]. Technology serves as the pivotal element, while business functions as the core. The integration of ICT technologies (e.g., radio, television, cell phones, computers, network hardware and software, satellite systems) has led to the development of a digital world characterized by awareness, connectivity, scenario-based intelligence, and automation. This digital transformation has the potential to enhance operational efficiency, redefine business models, and transform traditional management approaches. Consequently, enterprises can achieve continuous improvement in competitiveness [19]. As articulated by Scholar Vial (2019) [20], the term “digitalization” is defined as the process of enterprise digitalization that instigates substantial alterations in the characteristics of an entity through a synthesis of information technology, computing technology, communication technology, and connectivity technology with a view to enhancing the entity. Enterprises leverage restructured digital technologies to enhance their value creation models, leading to innovation in products and services, business model innovation, operational efficiency, and organizational performance enhancement, among other benefits [21]. In summary, this study posits that enterprise digitalization is the process by which an enterprise or organization transforms its traditional business into a digital business and uses digital technologies such as artificial intelligence, big data, cloud computing, blockchain, and 5G to improve business efficiency and quality.

From the perspective of the Job Demands-Resources (JD-R) theory, digitalization serves as a dual-force mechanism that influences employees’ work experience by altering job demands and job resources. On one hand, digital transformation introduces new job demands, such as increased information processing, constant connectivity, and role expansion, which can elevate stress and burnout. On the other hand, digital tools and automated systems can function as job resources that facilitate productivity, improve efficiency, and reduce task complexity when properly implemented. The balance between these factors determines employees’ psychological well-being and work performance [22].

Digitalization, particularly through social media engagement, can exacerbate role stress and contribute to higher rates of burnout [23]. In environments where digital tools significantly influence risk perceptions, this escalation in stress is especially pronounced [24]. The existing research on digitalization has identified two primary aspects to its definition. One such aspect is the technological support aspect, as evidenced by Andriole (2017) [25], who posited that digital transformation was enabled by information technology change. On the other hand, it is defined from the aspect of organizational change. For example, Wang et al., (2023) [26] posited that in the era of the digital economy, enterprises promote the deep integration of digital technology and organizational management through learning, innovation, integration, and other mechanisms to evolve to a digital system, which they termed ‘enterprise digitalization’. The rapid digitalization of work environments can introduce additional dynamic pressures, suggesting potential impacts on job stress. Drawing from the Conservation of Resources (COR) theory, employees experiencing digitalization-induced job stress may perceive the loss of valuable cognitive and emotional resources, further exacerbating job burnout and role overload. COR theory suggests that individuals strive to maintain and protect their resources, and when faced with excessive job demands, they experience stress and exhaustion, which aligns with the JD-R model’s assertion that excessive demands lead to resource depletion [27]. Therefore, digital tools not only change workplace operations but also significantly influence the levels of stress experienced by employees [28].

Warner and Waeger (2019) [29] proposed that businesses leverage technologies such as the Internet of Things (IoT) and artificial intelligence (AI) to facilitate transformation and optimize production processes, ultimately enhancing customer experience. Nevertheless, the digitalization of enterprises will unavoidably give rise to alterations in a multitude of domains, including enterprise production methods, production relations, and human resources [30]. The implementation of diverse digital technologies is likely to alter the methods and modes of enterprise production and management. This may consequently result in an increase in workload and work requirements [12]. In occupational psychology, role overload, role stress, and role conflict are distinct yet interrelated constructs. Role overload occurs when an individual is assigned more responsibilities than they can manage within a given timeframe, leading to cognitive and emotional exhaustion. This issue is intensified in digital work environments due to increased connectivity, blurred work-life boundaries, and constant availability demands [31]. Role stress is the overall strain experienced due to excessive job demands, uncertainty, or conflicting expectations. Role conflict, a component of role stress, arises when employees face incompatible or contradictory demands from different roles, regardless of workload intensity. While role overload reflects excessive quantitative demands, role conflict pertains to qualitative contradictions in responsibilities [32].

In digital workplaces, role overload and role conflict increasingly intersect. Employees must navigate overlapping digital and physical responsibilities, often facing competing demands from remote work expectations, multitasking, and digital surveillance pressures. These factors contribute to heightened job stress and reduced well-being. Understanding these distinctions is crucial for developing strategies to mitigate workplace stressors and enhance employee productivity. According to the JD-R model, excessive work demands can lead to a continuous depletion of employees’ physical and mental resources, thereby increasing employee role stress and role conflict [33]. This can be regarded as a specific manifestation of role overload.

Role overload was defined as the stress response experienced by employees when they assume a new job role [34]. Role overload refers to the situation in which an individual is required to complete an excessive number of tasks within a limited timeframe, forcing them to juggle multiple responsibilities simultaneously. While role overload is often viewed as a source of role stress, role stress and role conflict have been shown to exacerbate role overload, creating a cyclical cumulative effect [35]. When individuals experience ongoing role stress [36] (e.g., elevated job demands, time constraints, high performance expectations within the organization, etc.), they frequently find themselves compelled to assume additional responsibilities or extend their work hours, thereby amplifying role overload. For instance, employees in high-pressure environments may need to allocate more energy to addressing challenges that surpass their personal limits [37]. Role conflict pertains to the competing demands of multiple roles, compelling individuals to make trade-offs between roles that are not compatible. Research has demonstrated that individuals experiencing role conflict often require additional time and energy to reconcile the contradictions between different roles, which can further augment role burden [38]. For instance, an employee may need to meet both high performance demands from superiors and teamwork assignments, and these objectives may not be aligned, resulting in increased role burden. Role stress and role conflict lead to role overload, and overload further exacerbates stress and conflict, trapping individuals in a vicious cycle [39]. Individuals in high-responsibility occupations, such as doctors, teachers, and business managers, frequently experience chronic role overload due to the accumulation of role conflict and stress, which can have detrimental effects on their physical and mental health, as well as on their productivity [40]. In the contemporary business landscape characterized by enterprise digitalization, the phenomenon of role overload can be examined through three distinct lenses. Primarily, the digital transformation process necessitates that employees perpetually acquire and master novel technologies and instruments. This necessity, in turn, can precipitate a sense of inundation in individuals who feel compelled to maintain currency in their skill sets [41]. The advent of digital tools has concomitantly increased the demands for enhanced work efficiency, resulting in employees often feeling pressured by tasks that exceed the scope of traditional work models [42]. Secondly, in the course of implementing digital transformation, if an organization fails to distribute tasks equitably or imposes excessive demands on employees, it can readily give rise to the phenomenon of employee role overload [43]. The integration of digital tools and systems without a concomitant re-evaluation of work design has the potential to augment employees’ workloads, thereby precipitating a state of overload [8]. Thirdly, while digital technology has the potential to enhance work efficiency, its implementation may encounter challenges if the technology system is overly complex or does not align with employees’ actual needs. This can result in difficulties in utilization, increased work pressure, and the onset of role overload [44]. Enterprises’ overreliance on technology in the digital transformation process may result in the disregard of individual employee needs and psychological capacities, thereby leading to the exacerbation of role overload.

In the digital age, the continuous progression of information technology has engendered revolutionary changes in work processes and work styles. This transformation has given rise to role overload, manifesting in novel characteristics of employees’ work, which are predominantly evident in the following domains: The complexity of tasks borne by employees. The integration of digital technology has led to an increase in the volume of information processed by employees, the operation of more complex systems, and the necessity of mastering multiple skills [45]. This heightened complexity frequently results in task overload, necessitating extended time and energy expenditure on the part of employees to complete their assigned tasks [46]. Concurrently, employees are inundated with a deluge of digital information. The proliferation of digital technology, accompanied by the integration of big data, artificial intelligence, and other advanced technologies, has precipitated a surge in information. This deluge of data, in turn, has engendered a phenomenon of information overload. Employees are confronted not only with the sheer volume of information but also with the imperative to discern valuable data from it, thereby further augmenting their workload [47]. Furthermore, the boundaries between work and personal time have become increasingly indistinct. The proliferation of digital tools and mobile technology empowers employees to manage work tasks remotely and at any time. While this flexibility enhances work efficiency, it concomitantly imposes the pressure of perpetual availability, thereby exacerbating role overload [48]. Finally, employees are experiencing an increase in role conflicts. The rise in telecommuting and flexible work arrangements has led to employees facing competing demands not only from their professional roles but also from their familial and social responsibilities. This multifaceted role conflict can lead to role overload, as employees find themselves unable to effectively balance these various roles [49].Consequently, we propose the following hypothesis:

**H1:** Enterprise digitalization is positively related to workers’ role overload.

Enterprise digitalization enhances workplace efficiency by providing resources that streamline operations. However, it also introduces new demands that, if not carefully integrated, can paradoxically exacerbate role overload, thereby negatively impacting job satisfaction and employee well-being [43]. Burnout is a psychological state that can be observed in individuals who are subjected to prolonged periods of work-related stress. This state is characterized by three key symptoms: emotional exhaustion, a reduced sense of personal achievement, and deindividuation [50]. The specific manifestations of job burnout include the exhaustion and powerlessness of individuals in the case of ineffective coping with pressure, the negative, negative, and numb attitude of individuals toward service objects, and the reduced sense of personal achievement [51].

The process of enterprise digitalization will present a series of challenging tasks, including the development of new technologies and new platforms to ensure that digital technologies can be better integrated into enterprises’ production and operational practices [52].

According to extant research, the etiology of role burnout in the digital era can be summarized as follows: first, the dissolution of work boundaries. The advent of digital tools and mobile Internet has enabled employees to access work tasks at any time and from any location, thereby obfuscating the boundaries between work time and space. This state of “seamlessness” has been shown to result in heightened levels of work stress, emotional fatigue, and burnout [53]. Bamel (2022) [54] has identified a correlation between the use of digital technologies and the dissolution of traditional boundaries between work and personal life. In the long run, the convenience of digital communication has been found to necessitate that employees work beyond traditional business hours. This phenomenon engenders an organizational culture of perpetual availability, compelling employees to feel compelled to respond to work-related demands at all times.

This effectively extends working hours and reduces the separation between professional and private life. Individual efforts to enhance productivity, while laudable, are insufficient to address the systemic challenges confronting the organization [55]. Secondly, the presence of elevated expectations and pressure within the professional environment is a contributing factor. The rapid advancements of the digital age necessitate that employees perpetually adapt to novel technologies and methodologies. This necessitates not only the acquisition of novel digital competencies but also the execution of tasks within high-pressure environments, which can give rise to dehumanizing emotional responses and job dissatisfaction [56]. Thirdly, information overload is a salient issue. The digital environment has witnessed a substantial upsurge in information. Employees encounter challenges in effectively filtering and processing this deluge of information, which can result in information fatigue and emotional exhaustion [57]. Fourthly, the strengthening of performance monitoring. Enterprises utilize digital tools to conduct real-time monitoring and performance evaluation, thereby exerting an unseen pressure that can give rise to employee anxiety, which, in turn, can intensify role burnout [58].

In the context of digital transformation, the traditional theory of role burnout has gradually exposed some new problems and challenges. The rapid advancements in digital technology have profoundly impacted work styles and employee psychological well-being. In the era of digitalization, employee burnout exhibits distinctive characteristics, primarily the escalating technical pressure. The continuous updating of digital technology necessitates that employees constantly learn new technologies and software, which can lead to burnout when faced with the substantial pressure to update their skills [59,60]. Meanwhile, the workload has increased. The sheer volume of information in the digital age, coupled with the need for enhanced work efficiency, has led to a substantial increase in employees’ workloads. Prolonged periods of high-intensity work can lead to emotional fatigue and dehumanization. This phenomenon is further exacerbated by a perceived absence of work control, which can compound the challenges employees face [61,62]. Digital transformation has the potential to diminish employees’ sense of autonomy in their work. The integration of automated tools and intelligent systems within the work environment has been observed to diminish autonomy, thereby potentially resulting in a diminished sense of accomplishment [63]. Finally, the sense of social isolation increases. In digital work environments, particularly following the proliferation of remote work, employees’ social interactions are diminished, potentially leading to feelings of isolation and a lack of support from their teams, which can further exacerbate burnout [64]. As workers experience heightened stress, job satisfaction declines, which can culminate in burnout. Consequently, we propose the following hypothesis:

**H2:** Enterprise digitalization is positively associated with job burnout.

Digitalization impacts the sustainability of enterprise operations and necessitates effective work arrangements to prevent job burnout. By optimizing digital resources, it is possible to alleviate job demands and reduce role overload, enhancing workplace efficiency [65]. Role overload was defined as the psychological stress that occurs when the role receiver is unable to achieve the role goals by relying on their own resources, including knowledge, skills, and experience [66]. Role overload is a source of stress for employees when the demands of the role exceed the time and energy resources available to them [67]. In the context of enterprise digitalization, when enterprises require additional digitalization-related business functions (e.g., equipment maintenance, system operation, maintenance, etc.), employees are inevitably confronted with novel job content and roles [68]. Concurrently, Robinson’s (2005) [69] study indicated that a considerable proportion of workers are required to engage in additional information technology and digital transformation tasks beyond their regular duties. This entails the challenging task of promoting digitalization, which has the effect of increasing the content and intensity of their work, thereby exacerbating the sense of role overload experienced by employees.

Additionally, the process of enterprise digitalization necessitates the continual enhancement of one’s competencies. As enterprise digitalization continues to deepen and the rapid pace of technological change persists, the ever-changing applications of digital technology and challenging and escalating work are bound to emerge in rapid succession. Furthermore, the necessity to continually acquire new technological competencies can also serve as a source of occupational stress for employees, which may potentially result in role-loading in the absence of adequate support [70]. Role loading necessitates the exertion of physical or mental (cognitive and emotional) effort and skills, which may result in the expenditure of time or energy. According to the JD-R model, job demands serve as a crucial precipitating factor for exhaustion and burnout [71]. Exhaustion and burnout emerge when employees assume an excessive number of roles. Consequently, we propose the following hypothesis:

**H3:** Role overload mediates the relationship between digitalization and job burnout. Digitalization increases role overload, which subsequently leads to higher levels of job burnout.

As enterprises integrate digital tools, the increased efficiency and capabilities often come with heightened expectations and expanded job scopes, contributing to role overload. This overload, if not managed effectively, can lead to job burnout by overwhelming employees with excessive demands that exceed their capacity. Thus, understanding this intermediary role is essential for developing strategies that harness the benefits of digitalization while mitigating its potential downsides on employee well-being. The mediation model suggests that careful job design and resource allocation are crucial to prevent burnout in digitally transforming environments.

Perceived organizational support (POS) can be defined as employees’ overall perception of the extent to which the organization values their contributions and demonstrates care for their interests [72]. In accordance with the JD-R model, the diminution of job requirements resulting from the presence of job resources can serve to mitigate the incidence of job burnout [73,74]. In the context of enterprise digital transformation, the rapid superposition of digital technology knowledge, changes in workflow and content, and heavy system operation and maintenance work have emerged as key work requirements for employees. Organizational support can be utilized as a work resource to mitigate the adverse effects of job demands, such as role overload. When employees perceive organizational support, they are better equipped to cope with the demands and pressures of their roles. The implementation of supportive organizational practices, such as flexible working hours and mental health support, can assist employees in achieving a healthier work-life balance and mitigate the stress associated with role overload [75].

In the context of digital work environments, technostress is increasingly recognized as a significant factor contributing to employee role overload, which in turn acts as an intermediary leading to job burnout [76]. In a study conducted by Shanock and Eisenberger (2006) [77], managers were the subjects of analysis. The findings indicated that when organizations prioritize the personal health and work contributions of their employees, employees experience organizational incentives, which reduced the likelihood of emotional exhaustion. Concurrently, departments overseen by supportive leaders in select organizations would mitigate the adverse effects of role ambiguity on employees by granting them autonomy in their work and allowing them to participate in managerial decision-making [78]. Consequently, the following hypotheses were formulated:

**H4:** Perceived organizational support moderates both the direct relationship between digitalization and job burnout and the indirect effect through role overload, thereby influencing the causal chain from digitalization to burnout.

**H4a:** The moderating effect of perceived organizational support on the relationship between digitalization and burnout would be negative.

**H4b:** Perceived organizational support negatively would moderate the mediating effect of role overload between digitalization and burnout. Specifically, the negative relationship of digitalization on burnout via role overload would be attenuated when employees perceive high levels of organizational support.

**Fig 1:**
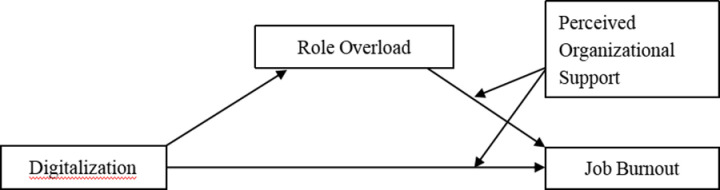
Research theoretical model.

Based on the above assumptions, the theoretical framework diagram of each variable in this study is formed (Fig 1).

## 3. Methods

### 3.1. Sample and data collection

The research process is reviewed and approved by the Office of Ethics and Compliance (OEC) of Chongqing Kelide New Material Technology Co., Ltd (CKNMT). Approval number is LLSC2022-06-1007. The distribution and collection of questionnaires adhered strictly to the guidelines of the Declaration of Helsinki and China’s Measures for Ethical Review of Life Science and Medical Research Involving Human Beings. The recruitment period for this study was conducted from January 1 to June 30, 2024, primarily through an online format. During this period, participants aged 18 years and above were recruited to complete the questionnaire, excluding minors from the study. The majority of participants provided verbal consent, which was audio-recorded by the researchers. During the questionnaire session, they were given an explanation of the study’s purpose, and completing the questionnaire indicated informed consent. Participants could withdraw at any time without any consequences.

Before submission, participants were prompted again to confirm their understanding and consent to the data’s use for research purposes. The questionnaire was anonymized, with no collection of private information, such as participants’ names. This study followed the principles of standardized informed consent, ensuring participants’ privacy rights and the right to withdraw. As a result, no additional signed consent forms were required.

A total of 278 completed questionnaires were retrieved, resulting in a response rate of approximately 92.7%. After excluding incomplete and ineligible responses, 250 valid questionnaires remained, yielding a validity rate of 83%. As illustrated in Table 1, the descriptive analysis table of the sample reveals that the largest proportion of employees falls within the 26–30 age range, constituting 36% of the total. Subsequently, employees within the 18–25 age range constitute 30.4% of the total sample. The age distribution trend of the questionnaire respondents is largely consistent with the research theme of this study. A total of 61.6% of the respondents had obtained a bachelor’s degree or higher. With regard to the distribution of respondents by industry, the highest proportion is represented by those working in manufacturing, at 18%.

**Table 1:** Descriptive statistics.

Type	Proportion	Type	Proportion
**Age**		**Occupation**	
18~25	30.40%	Employees of manufacturing companies	18.00%
26~30	36.00%	Administrative/Human Resources Management	9.00%
31~35	12.40%	Marketing/Sales/Commerce	7.20%
36~40	14.40%	Design practitioner	6.80%
Above 40	6.8%	Procurement	6.00%
**Education**		Service industry staff	6.00%
Junior high school and below	4.00%	Agriculture, forestry and Fisheries labor	6.00%
High school or equivalent	8.80%	Business manager	5.60%
Junior college	25.60%	Lawyer	5.60%
Bachelor’s degree	37.20%	Technical development engineer	6.00%
Postgraduate and above	24.40%	Finance/Accounting/Audit Staff	5.20%
**Type of enterprise**		Education	5.00%
Public enterprise	32.00%	Self-employed	4.00%
Domestic private business	38.00%	Scientific researcher	4.00%
Foreign-owned enterprises or Joint ventures	12.00%	Product operation	3.60%
Other types	18.00%	Medical and healthcare	2.00%

### 3.2. Measurements

The questionnaire scale formation method is primarily comprised of three steps. The initial step entails the utilization of a more established scale, developed by both domestic and international scholars. The second step involves the selection of ten employees from three information technology service enterprises and other pertinent locations, with the objective of conducting a preliminary interview survey to ascertain the clarity, comprehensibility, and practical applicability of the questionnaire items. The third step necessitates the engagement of several experts in the field for consultation. In accordance with the findings of the preliminary research and the recommendations of the experts, some of the less accessible and challenging clauses were removed or modified. The scales were scored on a five-point Likert scale, with 1 indicating complete non-conformity and 5 indicating complete conformity. The total reliability (Cronbach’ s α) of the scale was 0.841.

The digitalization measurement employs the organizational practices update questionnaire developed by Wang et al. (2012) [79]. Digital transformation can be defined as the integration of digital technologies into an organization, which results in changes to its work practices, processes, and so forth [80]. Organizational practices represent the most basic unit of analysis in the study of enterprise evolution and are identified as the primary driver of digital transformation [81]. In the digital era, the renewal of organizational practices is the foundation upon which enterprises can obtain competitive advantages through digital transformation [82]. Therefore, this study posits that the organizational practice update questionnaire is an efficacious instrument for measuring the digitalization of enterprises. The questionnaire comprises eight items, including the statement that ‘enterprises take the initiative to change their organizational structure and business model in order to adapt to the challenges of the digital age’. The questionnaire is a unidimensional one. To ensure the reliability and validity of the questionnaire, an exploratory factor analysis was conducted on the results. The results are shown in Table 2. Each item exhibited a factor load value greater than 0.5, and there was no cross-loading. The total explanation of the difference between factors was greater than 60%. The internal consistency reliability coefficient of the questionnaire was 0.959, indicating good structural validity (Table 2).

**Table 2:** Factor load, reliability and validity.

Variable	Items	Loading value	AVE	CR	α
Digitalization	OT1	0.814	0.667	0.941	0.959
	OT2	0.782			
	OT3	0.832			
	OT4	0.811			
	OT5	0.779			
	OT6	0.829			
	OT7	0.847			
	OT8	0.838			
Perceived organizational support	OS1	0.773	0.637	0.925	0.928
	OS2	0.77			
	OS3	0.803			
	OS4	0.779			
	OS5	0.811			
	OS6	0.813			
	OS7	0.834			
Job burnout	BO1	0.828	0.689	0.899	0.912
	BO2	0.824			
	BO3	0.843			
	BO4	0.825			
Role overload	RO1	0.863	0.713	0.881	0.886
	RO2	0.842			
	RO3	0.827			
The total reliability					0.841

The perceived organizational support questionnaire is derived from a related study conducted by Eisenberger and colleagues (1986) [83] and He (2020) [84]. Scholar He constructed an eight-item organizational support scale based on the scale developed by Eisenberger and employed it to assess the moderating impact of organizational support on the turnover intention of employees in service roles within enterprises. Structural validity represents the most critical validity index for testing the scale [85]. To enhance the questionnaire’s validity, this study adopted the approach of Xu and her colleagues (2014) [86] and removed one item with an insufficient factor load. The model fit degree after deletion is significantly superior to that prior to deletion. Ultimately, a questionnaire comprising seven items across three dimensions—adaptive support, employee value recognition, and relationship support—was constructed, with the primary items including ‘The enterprise cares about my welfare’. The internal consistency reliability coefficient of the questionnaire is 0.928, and it exhibits robust structural validity (see Table 2).

The Job Burnout Questionnaire is primarily based on the Burnout Measure Short Version(BMS) scale proposed by Malach-Pines (2005) [87], which simplifies the Burnout Measure(BM) scale by reducing the number of items from 21 to 10. These items encompass a range of negative emotional states and behaviors, including fatigue, disappointment, despair, depression, and self-denial. The reliability and validity of the questionnaire have been demonstrated through extensive verification in multiple occupational group measurements. In the preliminary survey, the respondents exhibited a low level of engagement with some of the questions. In this study, the item differentiation was calculated using the Pearson correlation method, as proposed by Zhang et al. (2010) [88] and the evaluation ability of each item was illustrated by the correlation degree between the item and the total score. Following the removal of items with a correlation coefficient of less than 0.4, four measurement items were ultimately identified, including ‘I always feel exhausted after work’. The internal consistency reliability coefficient of the burnout scale is 0.912, indicating good structural validity (see Table 2).

The questionnaire developed by Schaubroeck and his colleagues (2010) [89] was employed to assess the role overload of employees operating under conditions of asymmetric trust. The questionnaire was employed by Wang and Zhang (2016) [34] to assess the role overload of employees in an asymmetric trust context. The scale content encompassed items such as ‘the work requires me to do things beyond my limit.’ The internal consistency reliability coefficient of the role load scale was 0.886, indicating satisfactory structural validity (see Table 2).

Previous research has indicated that younger employees are at an elevated risk of experiencing job burnout, largely attributed to their limited work experience and the absence of effective coping strategies [90]. Additionally, occupational type has been identified as a factor influencing the prevalence of job burnout. Studies have shown that employees in the medical and education sectors are more likely to experience significant job burnout [91]. The disparate characteristics of organizational structures, such as those observed in the public and private sectors, can also give rise to differential effects on burnout [92]. A higher educational background has been linked to a lower risk of burnout [93]. Similarly, a study by Belinda Agyapong and his colleagues (2022) [94] found that age, education, occupation type, and organizational nature interact to predict job burnout. Consequently, this study controls for age, educational background, occupation type, and enterprise nature.

### 3.3. Reliability and validity analyses

#### 3.3.1. Common method biases.

In this study, the reliability and validity of the questionnaire were analyzed using the statistical software packages SPSS 23.0 and Stata 15.0. It is evident that respondents tend to answer in accordance with factors such as shared social expectations or similar emotional states. This may have an impact on the validity of the questionnaire. In the present study, the questionnaire was designed with the inclusion of anonymous responses and the reverse scoring of certain items. Concurrently, Harman’s one-factor test was employed to ascertain the presence of common method bias. The results of the exploratory factor analysis indicated the presence of five factors with trait roots exceeding 1 under the principal component analysis. The first factor accounted for 22.62% of the variance, which is less than the 50% threshold, while the total variance explained reached 77.13%, which is above the 60% threshold. The results demonstrate that common method bias has a negligible impact on the study’s findings.

#### 3.3.2. Reliability and validity tests.

In the present study, the reliability and validity of the questionnaire were examined using SPSS. The total reliability (Cronbach’s α) of the scale was 0.841, as demonstrated in Table 2. This value indicates that the questionnaire exhibited good reliability. Additionally, the measured reliability of each variable exceeded 0.7, suggesting that the questionnaire’s internal consistency was satisfactory. The AVE value of each variable exceeds 0.6, and the composite reliability is above 0.8, indicating that the convergent validity of the variables is within an acceptable range. Subsequently, the factor structure of the focal variables was examined through the use of confirmatory factor analysis procedures in Stata. The expected four-factor solution (digitalization, role overload, burnout, and perceived organizational support) demonstrated an acceptable fit with the data [chi-square (318) =536.495; CFI =0.961; SRMR =0.042; RMSEA = 0.052].

## 4. Results

### 4.1. Correlation analysis

The study employed the use of the statistical software packages SPSS and Stata for the purpose of data analysis. Table 3 presents the results of the correlation analysis, which indicated a significant and positive correlation between digitalization and role overload (*r* = 0.461, *p* < 0.01). A significant positive correlation was observed between digitalization and burnout (*r* = 0.509, *p* < 0.01). A significant and positive correlation was observed between digitalization and technology overload (*r* = 0.383, *p* < 0.01). A significant and positive correlation was observed between role overload and burnout (*r* = 0.373, *p* < 0.01). A significant and positive correlation was observed between technology overload and burnout (*r* = 0.189, *p* < 0.01). This provides further evidence to support all the hypotheses.

**Table 3:** Correlation analysis result.

Variable	*M*	*SD*	1	2	3	4	5	6	7	8	9
1. Age	3.070	1.261	1								
2. Education	3.690	1.059	-0.117	1							
3. Occupation	10.260	5.991	-0.143^*^	-0.151^*^	1						
4. Type of enterprise	2.140	1.036	0.319^**^	-0.024	0.006	1					
5. Digitalization	3.436	0.990	-0.033	-0.002	-0.008	-0.005	**(0.959)**				
6. Role overload	3.244	1.011	-0.019	-0.056	-0.104	0.001	0.461^**^	**(0.886)**			
7. Job burnout	3.668	0.980	-0.014	-0.062	-0.038	-0.113	0.509^**^	0.373^**^	**(0.912)**		
8. Technology overload	3.562	1.020	-0.036	0.051	-0.020	-0.026	0.383^**^	0.188^**^	0.189^**^	**(0.932)**	
9. Perceived organizational support	3.981	0.822	-0.075	-0.053	0.001	-0.026	0.470^**^	0.347^**^	0.381^**^	0.274^**^	**(0.928)**

*Note*: ^*^ *p*< 0.05; ^**^p< 0.01; N = 250; The Cronbach’s alpha is in parentheses.

### 4.2. Mediation effect test

This study employs structural equation modeling based on Mplus 7.0 to examine the path relationship between digitalization and other variables. As illustrated in Table 4, there is a notable positive correlation between digitalization and role overload (*β* = 0.468, *p* < 0.01). Consequently, hypothesis 1 is validated. Similarly, role overload can markedly elevate the level of burnout (*β* = 0.360, *p* < 0.01). Drawing 10000 random samples, we constructed 95% bias-corrected confidence intervals for the hypothesized indirect mediating effects. The results are presented in Table 4, which shows that the inclusion of control variables leads to a significant positive effect of enterprise digitalization on burnout, with upper and lower bounds not containing 0 within the 95% confidence interval. Therefore, the mediating effect is established, and H2 and H3 are supported. The findings indicate that role overload mediates the relationship between enterprise digitalization and job burnout. In conclusion, it can be stated that enterprise digitalization affects burnout through role overload.

**Table 4:** Bootstrap test results of the mediating effects.

	Value	Standard error	95% confidence interval	
Digitalization →role overload	0.468^**^	0.057		
Role overload →job burnout	0.360^**^	0.057		
Digitalization →job burnout (direct effect)	0.427^**^	0.064	0.302	0.551
Digitalization →role overload →job burnout (indirect effect)	0.077^*^	0.031	0.017	0.137

*Note*: ** *p* < 0.01, * *p* < 0.05, *N* = 250.

### 4.3. Further study: heterogeneity analysis

In the process of enterprise digitalization, there is often a discrepancy in the capacity for digital technology adaptation between younger and middle-aged, and older employees. Individuals in the younger age cohort typically demonstrate elevated digital literacy and greater acceptance of technology. Consequently, they are more inclined to adapt to novel technologies and workflows during the digital transformation process. Conversely, older employees may experience heightened stress when confronted with novel digital tools, potentially due to a comparatively limited digital skill set or diminished technological experience. This discrepancy in technological adaptability may result in older employees experiencing burnout at a higher rate than their younger counterparts [95,96]. According to data published by the National Bureau of Statistics of China, 64.6% of the total workforce in the information transmission, software, and information technology service industry is under the age of 35. The younger generation is inherently digitally competent. The younger generation has been socialized in an era defined by the pervasiveness of information technology. In comparison to their older counterparts, younger employees tend to demonstrate a higher level of digital literacy, which enables them to rapidly assimilate and adapt to emerging technologies. Consequently, investigating the phenomenon of burnout among younger and older employees in the context of corporate digitalization can enhance the reliability and generalizability of the findings of this study. In light of the aforementioned evidence, this study draws upon existing literature and employs a division node of 35 years of age. The younger employee group is thus defined as comprising individuals aged 18–35, while the older employee group is defined as comprising individuals aged 36 and above. The subgroups were grouped together to conduct a heterogeneous study.

As shown in Table 5, the older employee group (*β* = 0.528, *p* < 0.05) perceived higher burnout in enterprise digitalization than the younger employee group (*β* = 0.496, *p* < 0.01). The coefficient of burnout was similarly elevated in the cohort of older employees (*β* = 0.516, *p* < 0.05) relative to that observed in the younger employee group (*β* = 0.387, *p* < 0.05), with role overload identified as a mediating variable. This is primarily attributable to the fact that older employees may be deficient in the requisite digital competencies (digital literacy) and may exhibit a heightened apprehension and anxiety regarding novel technologies, which predisposes them to experience stress and burnout with greater frequency in the context of digitalization. Concurrently, older employees typically exhibit a heightened reliance on the established processes and work patterns of the organization. In the context of digitalization, these processes and work patterns may undergo significant transformation, necessitating that veteran employees undergo a period of relearning and adaptation to new methods. This can result in an increased psychological burden and role overload for these individuals.

**Table 5:** Heterogeneity analysis.

	Younger employees group		Older employees group	
Variable	Job burnout			
Age	0.067	0.057	-0.045^*^	-0.036^*^
	(0.905)	(0.796)	(-0.405)	(-0.305)
Education	-0.094^*^	-0.094^*^	-0.026	-0.019
	(-1.452)	(-1.491)	(-0.231)	(-0.159)
Occupation	-0.033	-0.022	-0.015	-0.009^*^
	(-0.474)	(-0.326)	(-0.14)	(-0.077)
Type of enterprise	-0.145	-0.155^*^	-0.094^*^	-0.092^*^
	(-2.119)	(-2.315)	(-0.855)	(-0.828)
Digitalization	0.496^**^	0.387^**^	0.528^*^	0.516^*^
	(7.745)	(5.430)	(4.895)	(4.260)
Job overload		0.229^*^		0.029^*^
		(3.197)		(0.216)
R^2^	0.257	0.294	0.197	0.434
△R^2^	0.277	0.040	0.331	0.038
N	197		53	

*Note*: ^*^ *p*< 0.05; ^**^p< 0.01; The t-value is in parentheses.

### 4.4. Moderating effects test

As indicated by the extant literature, perceived organizational support has been demonstrated to moderate the relationship between burnout and job satisfaction. This study examined the moderating effect of perceived organizational support on burnout in the context of enterprise digitalization, and the results are presented in Table 6. The path coefficient of perceived organizational support with control variables was 0.589, which was significant at the 0.05 confidence interval. Conversely, the path coefficient of the interaction term between enterprise digitalization and perceived organizational support was found to be significant (β = -0.922, p < 0.05), thereby suggesting a moderating effect of perceived organizational support on the relationship between enterprise digitalization and burnout. The findings of the data analysis provide substantial evidence in support of Hypothesis 4a.

**Table 6:** Results of moderating effect analysis.

Variable	Job burnout
Age	0.044 (0.764)
Education	-0.047 (-0.866)
Occupation	-0.058 (-1.045)
Type of enterprise	-0.120^*^ (-2.131)
Digitalization	1.071^**^ (3.777)
Perceived organizational support	0.589^**^ (3.163)
Digitalization × Perceived organizational support	-0.922^*^ (-2.330)
R^2^	0.298
△R^2^	0.040

*Note*.^**^ *p*<0.01, ^*^ *p*<0.05, The t-value is in parentheses, *N* = 250.

### 4.5. Moderated mediation effects test

Existing research has frequently employed the Process Macro method to rigorously test moderated mediation models, providing a robust framework for examining complex relationships between variables. In this study, we primarily draw inspiration from the methodological approach of Nguyen et al. [97,98], who utilized the Process Macro in their two-wave time-lagged study, Firms’ green knowledge sharing and tourists’ green electronic word-of-mouth intention: A two-wave time-lagged study of moderated mediation model. Nguyen’s research effectively applied this method to assess the mediating role of tourist attitudes in the relationship between firms’ green knowledge sharing and green electronic word-of-mouth intention, while also exploring moderating factors such as environmental consciousness. By adopting a similar approach, our study leverages the Process Macro (Model 15) to enhance the transparency and reliability of our findings, ensuring a comprehensive analysis of the moderated mediation effects linking digitalization, role overload, and job burnout.In line with the recommendations for robust estimation procedures, 10000 bootstrap samples were employed to generate 95% confidence intervals for all effects, thereby enhancing the statistical reliability of the findings.

The results show that digitalization not only exerts a direct, positive effect on burnout but also indirectly influences burnout by increasing role overload (Tables 7 and 8). First, the direct pathway from digitalization to burnout is moderated by perceived organizational support, as evidenced by the significant interaction term ‘Digitalization × POS’ (*β* = -0.1195, *p* < 0.01). When employees report higher levels of organizational support, the adverse effect of digitalization on burnout is substantially attenuated. This suggests that organizational resources, such as training, emotional support, and workload management, can buffer the strain associated with the introduction of new technologies and processes.

**Table 7:** Key path coefficients and moderating effects.

Path	Coefficient	SE
Digitalization →Role overload	0.468^**^	0.057
Role overload →Job burnout	0.360^**^	0.057
Digitalization →Job burnout	0.427^**^	0.064
Digitalization × POS →Job burnout	-0.119^**^	0.078
Role overload × POS →Job burnout	-0.071^*^	0.076

*Note*: ** p<0.01, * p<0.05, N = 250.

**Table 8:** Direct and indirect effects.

	POS	Effect	SE	LLCI	ULCI
Conditional direct effect of X on Y: Digitalization-> Job burnout	3.0229	0.4761	0.0971	0.2849	0.6672
	4.2857	0.3252	0.0683	0.1907	0.4596
	4.7143	0.2739	0.0863	0.1039	0.4439
Conditional indirect effects of X on Y: Digitalization-> Role overload-> Job burnout	3.0229	0.1022	0.0439	0.0242	0.1964
	4.2857	0.0598	0.0311	0.0012	0.1241
	4.7143	0.0454	0.0394	0.0336	0.1225

The indirect pathway ‘Digitalization → Role overload → Job burnout’ is also moderated by perceived organizational support (Tables 7 and 8). The interaction term ‘Role overload × POS’ (*β* = -0.0714, *p* < 0.05) indicates that role overload translates into burnout more strongly when POS is low. As POS increases from 3.0229 to 4.2857 and then to 4.7143, the indirect effect of digitalization on burnout via role overload decreases from 0.1022 to 0.0598 and 0.0454, respectively. These results imply that employees who perceive stronger support from their organizations are better able to cope with the expanded tasks and responsibilities brought about by digitalization, thereby reducing the likelihood of burnout.

## 5. Discussion and implications

### 5.1. Discussion

This study investigates the impact of digitalization on employee burnout, emphasizing the mediating role of role overload, age-based differences, and the moderating effect of perceived organizational support (POS). The findings both corroborate and extend prior research on digitalization-induced burnout, offering nuanced insights into its mechanisms and contextual factors.

Digitalization significantly induces role overload by increasing task complexity and time pressures among employees. This observation aligns with findings from Day et al. (2012) [99], who demonstrated that the integration of information and communication technologies (ICT) escalates work demands, leading to heightened stress. Similarly, Brown et al. (2014) [100] found that digital tools often expand job responsibilities, requiring employees to manage additional cross-functional tasks. This study extends these insights by showing that rapid digital adoption—such as implementing new platforms and analytics—further exacerbates role overload, particularly in fast-paced organizational transitions [101]. Such demands resonate with Job Demands-Resources (JD-R) theory, where excessive technological demands diminish employees’ resources, fostering strain [71].

Role overload mediates the relationship between digitalization and burnout, channeling the effects of sustained digital demands into psychological fatigue. This finding corroborates Maier et al. (2015) [102], who identified work overload as a critical pathway through which technology use contributes to exhaustion. Likewise, Suh and Lee (2017) [103] reported that constant connectivity and data-intensive tasks amplify emotional strain, a pattern this study confirms. Compared to Giorgia Bondanini and his colleagues (2020) [104], who broadly linked technostress to burnout, this research specifies role overload as the pivotal mechanism, offering a more precise explanatory framework. This mediation underscores how poorly managed digitalization can erode employee well-being despite its efficiency potential.Meanwhile, our findings align with prior research highlighting the role of psychological mechanisms in shaping behavioral outcomes. For instance, Nguyen et al.(2025) [105] demonstrated that psychological ownership and knowledge sharing serve as key drivers of sustainable tourist behavior. Similarly, in the workplace context, employees with a stronger sense of psychological ownership over digital tools or processes might perceive role overload as less burdensome, thereby reducing burnout.

Age significantly influences digitalization-induced burnout, with older employees experiencing higher levels than their younger counterparts. This result aligns with Morris and Venkatesh (2000) [106], who noted that older workers face greater challenges adapting to new technologies, resulting in elevated stress. However, it contrasts with findings from Wang. (2023) [22], who suggested younger professionals exhibit higher digital burnout due to over-reliance on technology. This study’s evidence of steeper learning curves and skill obsolescence anxiety among older employees enriches the discourse, highlighting a generational divide overlooked in prior work [106]. This discrepancy suggests that age-related responses to digitalization warrant further exploration.

Perceived organizational support (POS) effectively moderates the relationship between digitalization and burnout, mitigating its adverse effects. This finding echoes Rhoades and Eisenberger (2002) [107], who established that POS buffers workplace stressors by enhancing resource availability. Extending this, Dara (2025) [108] found that supportive interventions reduce technostress in digital contexts. The moderating role of perceived organizational support (POS) in our study resonates with findings from digital contexts beyond the workplace. For example, Nguyen et al.(2025) [109] found that knowledge sharing in digital spaces enhances sustainable engagement and electronic word-of-mouth in tourism, underscoring the importance of supportive mechanisms in driving positive outcomes. In our research, POS similarly attenuates the indirect effect of digitalization on burnout via role overload, suggesting that facilitating knowledge sharing could amplify employees’ resilience to technological demands. This parallel highlights a broader applicability of knowledge-sharing frameworks in mitigating strain across diverse settings, from tourism to organizational digitalization. This study advances these insights by demonstrating that POS not only directly alleviates burnout but also weakens the mediating impact of role overload, a dual effect less emphasized in previous research [110]. This moderation highlights the protective role of organizational resources in digital transitions [111].

### 5.2. Implications and suggestion

This study contributes to the existing literature on the negative consequences of enterprise digitalization by examining the impact of digital technologies on employee cognitive behavior. Currently, there is a paucity of research examining the potential adverse consequences of digital transformation and the utilization of digital technology. For example, Sai Yuan and his colleagues (2023) [112] posited that digital transformation will exacerbate the salary disparity between employees and senior executives, and intensify the internal inequity of employee compensation. Nevertheless, there has been a paucity of research examining the adverse effects of enterprise digitalization on employees’ psychology. In order to meet the needs of the digital economy era, the digitalization of enterprises may be a disruptive change, which presents challenges to the construction of enterprises’ organizations and personnel [113]. This study draws on the cognitive theory of organizational change to examine the potential implications of digitalization on employees’ working methods. It highlights the possibility that the negative cognition of organizational change may result in role overload and job burnout, thus broadening the understanding of enterprise digitalization from the perspective of employees’ cognitive behavior.

In addition, the findings of this study indicate that the process of enterprise digitalization will inevitably result in the emergence of negative emotional states among employees, including job burnout. It is currently accepted that digital transformation may result in short-term challenges for the labor market and human resource management [114]. Nevertheless, following an in-depth examination, this study posits that in the immediate term, enterprise digitalization will inherently elevate work intricacy and workload, precipitating an increase in role burden and job burnout. In the long term, enterprise digitalization will give rise to an ‘always on call’ organizational culture, which will result in employees experiencing prolonged feelings of role overload and job burnout. Moreover, the findings of this study indicate that enterprise digitalization may result in digital overload and a productivity paradox [115]. Specifically, digital tools designed to enhance employee productivity can generate a vast amount of information, which may ultimately diminish productivity and increase workload over time, leading to role overload.

The study also revealed that younger and older employees exhibited disparate perceptions of burnout in the context of digitalization within organizations. This can be attributed to four main factors. Primarily, older employees may encounter greater challenges in adapting to the technological requirements of digital transformation due to their extensive experience in traditional work environments. Research indicates that older employees may lack the requisite digital skills (digital literacy) and exhibit heightened apprehension and anxiety about novel technologies, which renders them more susceptible to stress and burnout in the context of digital transformation [116]. Conversely, new employees typically possess superior digital competencies and adaptability. Secondly, older employees typically exhibit a greater reliance on established processes and work patterns within the organization. In the context of digital transformation, these processes and work patterns may undergo significant changes, necessitating that veteran employees relearn and adapt to new methods, which can increase their psychological burden [117]. In contrast, new employees lack this sense of dependency and are more likely to align their work patterns with digital processes, reducing the likelihood of burnout during the transition. Moreover, as an enterprise’s digitalization process progresses, veteran employees may perceive a mismatch between their professional skills and the evolving organizational needs, or even face the possibility of redundancy. This sense of occupational insecurity can result in a reduction in commitment to one’s work, thereby precipitating burnout. Furthermore, this resistance is also likely to exacerbate the sense of burnout. This argument is supported by the findings of several scholars, including Nkomo and Kalisz (2025) [118] and Nabeel Rehman et al.,(2021) [119].

In the contemporary digital economy, enterprises have widely adopted digital technologies such as blockchain, artificial intelligence, and the Internet of Things to improve the efficiency of production and operation. These technologies have facilitated the digital transformation and optimization of enterprise production, operations, and management, achieving cost reductions, quality improvements, and efficiency gains. However, the proliferation of digital work has concomitantly given rise to challenges such as information overload, perpetual online presence, and heightened work intensity. These factors have led to an escalation in psychological distress and the risk of burnout among employees. Consequently, there is an evident necessity to undertake research on employee mental health in the context of enterprise digitalization.

First, the promotion of digital wellness programs and enhancement of organizational support systems is imperative. As organizations progress in their digital transformation, it becomes imperative for them to proactively implement digital wellness programs. This proactive approach ensures that employees are able to maintain a healthy balance between productive work and mental well-being. The integration of digital health management tools is also paramount. The utilization of artificial intelligence and big data facilitates the analysis of employees’ work habits, the monitoring of workload, and the provision of intelligent mental health support, including stress management advice and personalized rest reminders. The provision of regular mental health services is also paramount. The establishment of employee counseling rooms, the introduction of EAP (Employee Assistance Program), and the provision of regular counseling, meditation training, and mental health seminars are recommended to assist employees in developing positive coping mechanisms. Optimizing workload distribution is also paramount. Organizations should employ data analysis tools to optimize task distribution, ensuring that employees are not overwhelmed, which can lead to anxiety and burnout, while allocating sufficient time for rest and adjustment. The digitalization of health incentives is also recommended. Enterprises can establish health incentive mechanisms, such as encouraging employees to engage in regular physical activity, participate in health-promoting activities, and utilize digital platforms to record and share their health status, with the objective of enhancing employees’ awareness of their own health.

Secondly, it is imperative to elucidate the boundaries between professional and personal domains to mitigate the onset of “digital fatigue.” The proliferation of remote work, digital collaboration tools, and real-time communication platforms has engendered a pervasive blurring of the distinction between professional and personal domains, thereby precipitating an escalation in the prevalence of “always-on” employees. Enterprises must establish a clear “digital disconnection” policy to mitigate this issue. Such a policy could include a directive that work emails and messages be left unanswered during non-working hours. Furthermore, enterprises could implement an automatic message reminder system to ensure employees are aware of the importance of disengaging from work notifications outside of working hours. The implementation of a flexible office model is also recommended. This model empowers employees to select work hours that align with their individual pace, while ensuring that telecommuters receive equivalent rest and vacation benefits as their office-based counterparts. Enterprises can organize activities such as No Screen Day and outdoor group building activities to encourage employees to reduce their reliance on electronic devices and promote physical and mental health.

In addition, it is imperative to reinforce the training of managers to enhance their capacity to identify and address instances of burnout. It is imperative that middle and senior managers of enterprises not only assume responsibility for business promotion in the context of digital transformation, but also possess the capacity to identify employee burnout and implement timely interventions. Consequently, enterprises should prioritize the development and implementation of training programs designed to enhance the identification of burnout among employees. These programs should be conducted on a regular basis to ensure the continuous dissemination of knowledge and skills related to recognizing and responding to burnout. Furthermore, enterprises should assist managers in identifying stress signals exhibited by employees, such as decreased concentration, depressed mood, lack of work motivation, and frequent absenteeism. The establishment of a feedback mechanism for mental health is also crucial. Furthermore, it is imperative for managers to engage in regular, one-on-one communication with their employees, thereby fostering a conducive environment for the articulation of concerns regarding the challenges associated with digital transformation. In addition, managers must be equipped with the necessary resources to provide targeted support. Furthermore, the implementation of a flexible adjustment mechanism is crucial. For employees deemed to be at high risk of burnout, companies can provide flexible working arrangements, such as short-term leave, job rotation, or psychological counseling support to help employees return to work. Furthermore, cultivating a corporate culture that is psychologically safe is imperative. Furthermore, encouraging managers to cultivate an atmosphere of openness, trust, and support within their teams is paramount. By fostering such an environment, employees feel empowered to express their stress and difficulties without fear of retribution or negative evaluation, thereby promoting a psychologically safe corporate culture.

Finally, it is imperative to strike a balance between the objectives of digitalization and the well-being of employees to establish a sustainable development model. Throughout the process of digitalization, enterprises must explicitly communicate that the primary objective of digitalization encompasses not only enhancing operational efficiency and competitiveness, but also ensuring that employees possess the capacity to adapt to the emerging technological milieu in a healthy and stable manner. Enterprises, therefore, must consider the psychological expectations of employees when promoting digitalization, formulating a reasonable development path for digitalization that avoids both too rapid technological change and excessive pressure, both of which can lead to employee anxiety and resistance. It is imperative to avoid an exclusive emphasis on technological progress, while neglecting the adaptability of employees, to ensure that the introduction of new technologies and employee training are synchronized, thereby reducing occupational pressure due to skills mismatch. Furthermore, enterprises should fortify their organizational support during digital transformations and establish interdepartmental communication channels to ensure employees receive timely assistance when confronted with challenges.

### 5.3. Limitations

In regard to the selection of samples, it can be observed that, given the inherently high-tech nature of digitalization, the employees included in this study generally possess a high level of knowledge and education. Therefore, the findings of this study may not be readily generalizable to employees in other industries. In terms of measurement methods, this study primarily relies on employees’ subjective perceptions when assessing variables such as enterprise digitalization and job burnout. Consequently, external normative pressures may exert an influence on employees’ responses, which could result in a discrepancy between their stated views and the actual situation.

Additionally, as a cross-sectional study, this research is unable to account for changes in attitudes, behaviors, or conditions that may have occurred before or after the survey period. Moreover, the accuracy of the data is contingent upon the participants’ ability to recall past events and experiences with precision. This may result in recall bias, whereby respondents may forget, exaggerate, or underestimate aspects of their perceptions. These factors may restrict the broader applicability of the findings.

It would be beneficial for future research to consider combining enterprise revenue data post-digitalization, keyword text analysis from annual reports on digitalization, employee interviews, and other sources of data to triangulate findings, thereby ensuring greater measurement accuracy and research rigor. Furthermore, given the intrinsic dissimilarities in production techniques between conventional manufacturing and information technology service sectors, future research could undertake a more comprehensive examination of three pivotal areas: firstly, the impact of digitalization on employees from diverse cultural backgrounds to ascertain the influence of cultural elements on digitalization outcomes; secondly, the investigation of industry-specific variations in digitalization to identify optimal practices across sectors; and thirdly, the exploration of optimal technological solutions for enterprise digitalization through the utilization of cross-disciplinary research methodologies to identify pertinent tools and platforms.
